# Long noncoding RNA/circular RNA regulates competitive endogenous RNA networks in rheumatoid arthritis: molecular mechanisms and traditional Chinese medicine therapeutic significances

**DOI:** 10.1080/07853890.2023.2172605

**Published:** 2023-03-11

**Authors:** Jianting Wen, Jian Liu, Lei Wan, Fanfan Wang

**Affiliations:** a Department of Rheumatology and Immunology, First Affiliated Hospital of Anhui University of Chinese Medicine, Hefei, Anhui, China; b Institute of Rheumatology, Anhui Academy of Chinese Medicine, Hefei, Anhui, China; c Key Laboratory of Xin’an Medicine of the Ministry of Education, Anhui University of Chinese Medicine, Hefei, Anhui, China; d Department of Internal Medicine Application Foundation Research and Development, Anhui Province—Key Laboratory of Modern Chinese Medicine, Hefei, Anhui, China

**Keywords:** lncRNAs, circRNAs, ceRNA, rheumatoid arthritis, molecular mechanisms, therapeutic implications

## Abstract

Rheumatoid arthritis (RA) is a systemic and autoimmune disease that is mainly featured abnormal fibroblast-like synoviocyte (FLS) proliferation and inflammatory cell infiltration. Abnormal expression or function of long noncoding RNAs (lncRNAs) and circular RNAs (circRNAs) are closely related to human diseases, including RA. There has been increasing evidence showing that in the competitive endogenous RNA (ceRNA) networks, both lncRNA and circRNA are vital in the biological functions of cells. Nevertheless, the exact mechanism of ceRNA in RA remains to be investigated. Herein, we summarized the molecular potencies of lncRNA/circRNA-mediated ceRNA networks in RA, with emphasis on the phenotypic regulation of ceRNA in the progression of RA, including regulation of proliferation, invasion, inflammation and apoptosis, as well as the role of ceRNA in traditional Chinese medicine (TCM) in the treatment of RA. In addition, we also discussed the future direction and potential clinical value of ceRNA in the treatment of RA, which may provide potential reference value for clinical trials of TCM therapy for the treatment of RA.

**Key messages**
- Long noncoding RNA/circular RNA can work as the competitive endogenous RNA sponge and participate in the pathogenesis of rheumatoid arthritis.
- Traditional Chinese medicine and its agents have shown potential roles in the prevention and treatment of rheumatoid arthritis via competitive endogenous RNA.

## Introduction

Rheumatoid arthritis (RA) is an inflammatory and destructive disease with clinical manifestations of primarily symmetrical polyarthritis and extra-articular injury [1,2]. It eventually contributes to joint deformity and disability, seriously reduces the patient’s quality of life and affects social participation, which is considered as ‘Deathless cancer’ [3–6]. The onset of RA has no age limitation with a high incidence, and the age of incidence is mainly 40–60 years, and the number of female RA patients is two to three times of male RA patients [3,7]. Currently, the exact pathogenesis of RA remains unknown. This chronic autoimmune disease with a high disability rate, is easy to attack repeatedly and is difficult to cure [8]. Meanwhile, the long treatment cycle of this disease brings a huge financial burden to the patient’s families and society. The current treatment approaches for RA are based on glucocorticoids, non-steroidal anti-inflammatory drugs, traditional anti-rheumatic drugs, as well as biological agents [9,10]. Nevertheless, improper drug administration may cause cardiovascular system damage, liver and kidney dysfunction, gastrointestinal discomfort and other adverse reactions [11]. Moreover, the expensive biologic agents weaken the immune system and thus may increase the risk of infections [12].

Fibroblast-like synoviocytes (FLSs) are key effector cells in RA and are regarded as possible therapeutic targets for RA [13,14]. FLS is a crucial player in RA pathogenesis, exhibiting diverse invasive features, including apoptosis resistance, hyperproliferation, enhanced invasiveness, as well as secretion of inflammatory mediators [15]. On the one hand, RA-FLS can synthesize and secrete matrix metalloproteinases to erode the cartilage, causing inflammatory cell infiltration into the involved joints and chronic cartilage destruction [16]. On the other hand, activated FLSs secrete many chemokines, growth factors and pro-inflammatory cytokines to promote disease severity [17]. In addition, the defective apoptosis of FLSs can result in synovial excessive proliferation, pannus formation and progressive joint destruction with irreversible loss of articular function [18]. Moreover, oxidative stress exhibits a positive relation with inflammation and promoted joint destruction in RA patients [19,20]. It is suggested that the proliferation, invasion, inflammation and apoptosis of RA-FLS are potential mechanisms in RA.

Noncoding RNAs (ncRNAs) account for more than 98% of the human genome and play an important role in gene expression and regulation, including Long noncoding RNAs (lncRNAs), circular RNAs (circRNAs), microRNAs (miRNAs), transcribed pseudogenes [21]. miRNAs (20–200 nucleotides) function by binding to complementary sequences in the 3′-untranslated region (UTR) of their target mRNAs, thereby triggering translational repression of transcripts or mRNA degradation [22]. lncRNAs (more than 200 nucleotides) usually do not encode proteins and act as transcriptional regulators [21]. circRNAs are endogenous ncRNAs lacking the 5′ and 3′ ends, and their loop-like structure gives them a higher stability [23]. The sequence of a pseudogene is usually similar to the corresponding gene, but is at least partially lost, such as not encoding a protein or encoding a protein without function [24]. Long noncoding RNAs (lncRNAs), circular RNAs (circRNAs), microRNAs (miRNAs), as well as transcribed pseudogenes, correlate with many diseases, including RA belonging to autoimmune diseases [25–27]. Although many studies have provided evidence that both lncRNAs and circRNAs become a research hotspot in RA through their functions in many life activities [28,29]. However, at the sequence level, lncRNAs and circRNAs are poorly conserved across species.

**Figure 1.**
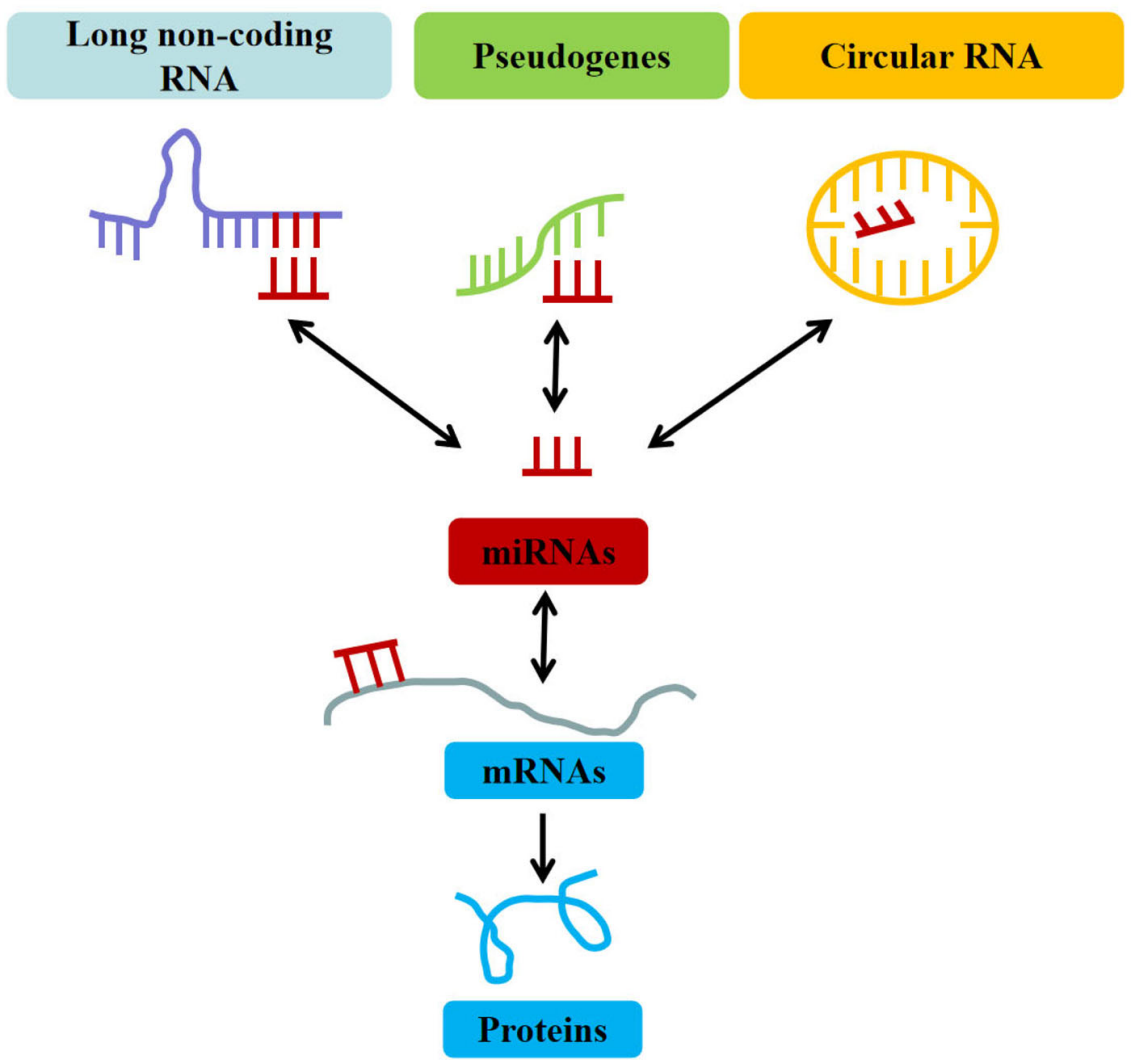
The concept and types of the competitive endogenous RNAs (ceRNAs).

In 2011, Salmena et al. proposed a competitive endogenous RNA (ceRNA) hypothesis describing that lncRNA/circRNA competes with protein-coding mRNA to bind miRNA [30]. It emphasizes that lncRNAs/circRNAs act as miRNA sponges that protect target mRNAs from inhibition by sequestering specific miRNAs [30] (Figure 1). However, due to the limitation of current experimental approaches, this challenging area of ceRNA research is still in its infancy, and there are still many aspects to be improved and some issues to be solved. Fortunately, recent research has shown that ceRNAs participate in several diseases, including cardiovascular and cerebrovascular diseases [31], nervous system diseases [32], respiratory diseases [33], immune system diseases [34,35] and malignant tumours [36,37].

Given the important role of ceRNAs in RA, this review concentrated on the lncRNAs/circRNAs-mediated ceRNA networks to figure out the potential functions of lncRNAs/circRNAs in modulating RA. Besides, we systematically summarized the functions of the lncRNA/circRNA-miRNA-mRNA axis in proliferation, invasion, inflammation and apoptosis in different cell lines and traditional Chinese medicine (TCM) intervention effect of ceRNAs in RA treatment (Table 1). Moreover, we probed into the challenges and therapeutic implications of ceRNAs in RA.

**Table 1.** Research involving the long noncoding RNA/circular RNA (lncRNA/circRNA)-mediated the competitive endogenous RNA (ceRNA) networks in rheumatoid arthritis (RA).

Reference	LncRNA/CircRNA	Model	Pathophysiological mechanism	Axis	Effects
Wang et al. [38]	LncRNA LINC-PINT	RA-FLS	Proliferation, invasion, and apoptosis	LncRNA LINC-PINT/miR-155-5p/SOCS1	LncRNA LINC-PINT contributed to the progression of RA *via* the miR-155-5p/SOCS1
Zhao et al. [39]	LINC01197	FLS of RA model mice	Inflammation	LINC01197/miRNA-150/THBS2	Downregulated LINC01197 contributed to reduced inflammation *via* the miR-150/THBS2 axis
Yang et al. [40]	LncRNA GAS5	RA-FLS	Proliferation, inflammation, and apoptosis	LncRNA GAS5/miR-222-3p/Sirt1	Silencing of lncRNA GAS5 led to promoted apoptosis and impeded proliferation and inflammation *via* the miR-222-3p/Sirt1 axis
Li et al. [41]	LncRNA MEG3	RA-FLS	Proliferation, inflammation, and apoptosis	LncRNA MEG3/miR‐141/AKT/mTOR	Reduced lncRNA MEG3 contributed to ameliorated proliferation and inflammation *via* modulating the miR‐141/AKT/mTOR axis
Wang et al. [42]	Linc0238	RA-FLS	Proliferation, invasion, inflammation, as well as apoptosis	Linc0238/miR-590-5p/MAP2K3	Suppression of Linc02381 expression resulted in suppression of RA progression *via* mediating the miR-590-5p/MAPK axis
Wang et al. [43]	LncRNA PVT1	RA-FLS, syno*via*l tissues of RA rats	Inflammation and apoptosis	LncRNA PVT1/miR-543/SCUBE2	LncRNA PVT1 was regarded as a miR-543 ceRNA to mediate SCUBE2 and suppress apoptosis
Yan et al. [44]	LncRNA HIX003209	RA-PBMCs, RA macrophages	Inflammation	LncRNA HIX003209/miR-6089/TLR4/NF-κB	LncRNA HIX003209 acted as a miR-6089 ceRNA to modulateTLR4/NF-κB expression, thereby exacerbating inflammation
Su et al. [45]	lncRNA HAND2-AS1	exosomal, MH7A, RA syno*via*l tissues	Proliferation, invasion, inflammation, as well as apoptosis	lncRNA HAND2-AS1/miR-143-3p/ TNFAIP3/NF-κB	Upregulation of exosomal lncRNA HAND2-AS1expression led to downregulated miR-143-3p, and enhanced TNFAIP3, further aggravating RA progression
Fan et al. [46]	LOC100912373	RA-FLS	Proliferation	LOC100912373/miR-17-5p/PDK1	LOC100912373 promoted the proliferation of RA-FLS *via* the miR-17-5p/ PDK1 axis
Wang et al. [47]	LINC00152	RA-FLS	Proliferation and apoptosis	LINC00152/miR-1270/FOXM1	Silenced LINC00152 contributed to the inhibited proliferation and stimulated apoptosis of RA-FLSs *via* the FOXM1/Wnt/β-catenin axis
Yang et al. [48]	LncRNA ZFAS	RA-FLS	Proliferation, apoptosis, inflammatory response, as well as autophagy	LncRNA ZFAS/miR-2682-5p/ADAMTS9	Knockdown of lncRNA ZFAS1 resulted in suppress RA progression *via* the miR-2682-5p/ADAMTS9 axis
Qing et al. [49]	LncRNA OIP5-AS1	RA syno*via*l tissue, RA-FLS	Proliferation, apoptosis and inflammation	LncRNAOIP5-AS1/miR-448/TLR3/NF-κB	Downregulating lncRNA OIP5-AS1 expression promoted apoptosis and suppressed proliferation and inflammation *via* regulation of the miR-448-1p/TLR3/NF-kB axis in RA patients
Rao et al. [50]	LncRNA NEAT1	(PBMC)-derived exosomes (exos) of RA	Proliferation and inflammatory response	LncRNA NEAT1/miRNA-23a/MDM2/SIRT6	The lncRNA NEAT1/miR-23a/MDM2 axis inhibited SIRT6 expression and promoted proliferation and inflammatory response
Mo et al. [51]	LncRNA GAPLINC	RA-FLS	Proliferation, invasion and inflammation	LncRNA GAPLINC/miR-382-5p/miR-575	Suppressing lncRNA GAPLINC expression retarded RA progression *via* the miR-382-5p/miR-575axis
Wang et al. [52]	LncRNA NEAT1	RA syno*via*l tissues, RA-FLS	Proliferation, invasion, inflammation, as well as apoptosis	LncRNA NEAT1/miR-410-3p/YY1	LncRNA NEAT1/miR-410-3p/YY1 axis functions in RA progression
Zhang et al. [53]	LncRNA NR-133666	RA-FLS	Proliferation and invasion	LncRNA NR-133666/miR-133c/MAPK1	LncRNA NR-133666 promoted proliferation and invasion through the miR-133c/MAPK1 axis
Zhu et al. [54]	lncRNA BZRAP1‐AS1	RA-FLS	Proliferation, inflammation and apoptosis	lncRNA BZRAP1‐AS1/miR‐1286/COL5A2	Suppressing lncRNA BZRAP1‐AS1 expression alle*via*ted proliferation and inflammation and promote apoptosis in RA through the miR‐1286/COL5A2 axis
Jiang et al. [55]	lncRNAS56464.1	FLS of RA model rat	Proliferation	lncRNAS56464.1/miR-152-3p/Wnt	LncRNAS56464.1 promoted proliferation by sponging miR-152-3p, thus downregulating Wnt expression
Ye et al. [56]	LncRNA CASC2	RA-FLS	Inflammation and apoptosis	LncRNA CASC2/miR-18a-5p/BTG3	LncRNA CASC2 sponges miR-18a-5p to aggravate inflammation and suppress apoptosis by upregulating BTG3 expression
Fu et al. [57]	LncRNA OSER1-AS1	RA-FLS	Proliferation, inflammation and apoptosis	LncRNA OSER1-AS1/miR-1298-5p/E2F1	LncRNA OSER1-AS1 reduced inflammation and promoted apoptosis *via* the miR-1298-5p/E2F1 axis
Liu et al. [58]	LncRNA XIST	RA-FLS	Proliferation and apoptosis	LncRNA XIST/miR-126-3p/NF-κB	Reduced lncRNA XIST led to reduced inflammation and promoted apoptosis through the miR-126-3p/NF-κB axis
Wang et al. [59]	LINC00665	MH7A	Proliferation, inflammation and apoptosis	LINC00665/miR-122-3p-3p/EIF2AK1	Knockdown of LINC00665 resulted in inhibiting RA progression *via* elevating miR-122-3p and decreasing EIF2AK1
Fu et al. [60]	lncRNA-H19	MH7A	Proliferation, inflammation and apoptosis	lncRNA-H19/miR-124a/CDK2/MCP-1	LncRNA-H19 aggravated RA-FLS proliferation by mediating miR-124a level
Zhuo et al. [61]	LncRNA ZNF667-AS1	RA syno*via*l tissues, RA-FLS	Proliferation and inflammation	LncRNA ZNF667-AS1/miR-523-3p/JAK/STAT	LncRNA ZNF667-AS1 was protective in RA by functioning as a miR-523-3p ceRNA to further target the JAK/STAT pathway
Zhang et al. [62]	lncRNA GAS5	RA-FLS	Proliferation and apoptosis	lncRNA GAS5/miR-361-5p/PDK4	lncRNA GAS5 was protective in RA-FLS *via* the miR-361-5p/PDK4 axis
Yao et al. [63]	LncRNA HOTTIP	Exosomes, RA-FLS	Proliferation and inflammation	LncRNA HOTTIP/miR-1908–5p/STAT3	Knockdown of lncRNA HOTTIP resulted in protection against RA *via* binding to miR-1908–5p to regulate STAT3 expression
Peng et al. [64]	LncRNA GAS5	RA syno*via*l tissues, RA-FLS	Proliferation, migration, invasion, inflammation, as well as apoptosis	LncRNA GAS5/miR-128-3p/HDAC4	Overexpression of lncRNA GAS5 caused aggravating RA progression through the miR-128-3p/HDAC4 axis
Zhang et al. [65]	LncRNA SNHG14	RA-FLS	Proliferation and inflammation	LncRNA SNHG14/miR-17-5p/MINK1/JNK	LncRNA SNHG14 impacted proliferation and inflammation by controlling the miR-17-5p/MINK1-JNK axis in RA
Wang et al. [66]	lncRNA RNA XIST	osteoblasts	Proliferation and inflammation	lncRNA RNA XIST/let-7c-5p/STAT3	LncRNA XIST aggravated proliferation and inflammation by serving as a ceRNA for let-7c-5p to target STAT3
Wang et al. [67]	LncRNA NEAT1_1	RA-FLS	Proliferation and invasion	LncRNA NEAT1_1/miR-221-3p/uPAR	LncRNA NEAT1_1 aggravated proliferation and invasion by controlling the miR-221-3p/uPAR axis
Zhao et al. [68]	LncRNA FOXD2-AS1	RA-FLS	Proliferation and invasion	LncRNA FOXD2-AS1/miR-331-3p/PIAS3	LncRNA FOXD2-AS1 strengthened proliferation and invasion capabilities by modulating the miR-331-3p/PIAS3 axis in RA
Zheng et al. [69]	LncRNA RNA ZFAS1	MH7A	Proliferation and apoptosis	LncRNA RNA ZFAS1/miR-296-5/MMP-15	LncRNA ZFAS1 exacerbates proliferation and suppresses apoptosis by modulating the miR-296-5p/MMP-15 axis
Zhong et al. [70]	Hsa_circ_0088036	RA-FLS	Proliferation and migration	Hsa_circ_0088036/miR-140-3p/SIRT1	Hsa_circ_0088036 sponged miR-140-3p to promote proliferation, migration and survival by decreasing SIRT1 expression
Li et al. [71]	circFADS2	chondrocytes	Apoptosis	circFADS2/miR-498/mTOR	CircFADS2 sponged miR-498 to inhibit apoptosis and survival through enhancing mTOR level
Zhi et al. [72]	Circ_AFF2	RA-FLS	Proliferation and inflammatory response	Circ_AFF2/miR-375/TAB2	Knockdown of circ_AFF2 resulted in reduced proliferation and inflammatory reaction *via* regulation of the miR-375/TAB2 axis
Cai et al. [73]	Circ_0088194	RA-FLS	Invasion and migration	Circ_0088194/miR-766-3p/MMP2	Circ_0088194 promoted the invasive and migratory capacities by the miR-766-3p/MMP2 axis
Chen et al. [74]	circ-PTTG1IP	RA-FLS	Proliferation, migration, invasion, as well as inflammatory response	Circ-PTTG1IP/miR-671-5p/TLR4	Circ-PTTG1IP promoted inflammatory response and biological functions of RA-FLS by the miR-671-5p/TLR4 axis
Hao et al. [75]	circ_0008360	RA syno*via*l tissues, RA-FLS	Proliferation, migration, inflammation, as well as apoptosis	Circ_0008360/miR-135b-5p/HDAC4	Circ_0008360 sponged miR-135b-5p to mediate DAC4 level, further impeded inflammation and biological functions in RA
Geng et al. [76]	Circ_0088036	RA-FLS	Proliferation, inflammation and apoptosis	Circ_0088036/miR-326/FZD4	Circ_0088036 silencing led to the restricted proliferation and inflammation reaction and diminished apoptosis *via* the miR-326/FZD4 axis
Li et al. [77]	circ_0130438	RA-FLS	Proliferation, migration, invasion, as well as inflammation	Circ_0130438/miR-130a-3p/KLF9	Circ_0130438 enhanced RA progression by the miR-130a-3p/KLF9 pathway
Wang et al. [78]	Circ_0088036	RA-FLS	Proliferation and inflammation	Circ_0088036/miR-1263/REL	Downregulation of circ_0088036 expression resulted in reduced RA-FLS proliferation and inflammation *via* the REL/NF-κB pathway activation and interaction with miR-1263
Qu et al. [79]	Circ-AFF2	RA-FLS	Proliferation, inflammatory response, migration, as well as invasion	Circ-AFF2/miR-650/CNP	Circ-AFF2 aggravated inflammatory response and biological functions by elevating CNP level *via* miR-650 sponging
Li et al. [80]	CircASH2L	RA-FLS	Proliferation, migration and inflammation, as well as apoptosis	CircASH2L/miR-129-5p/HIPK2	Inhibition of circASH2L reduced biological behaviors and inflammation *via* miR-129-5p/HIPK2 pathway
Luo et al. [81]	CircMAPK9	RA-FLS	Proliferation, migration and inflammation, as well as apoptosis	CircMAPK9/miR-140-3p/PPM1A	Inhibition of circMAPK9 possibly ameliorated the progression of RA by the miR-140-3p/PPM1A axis
Wang et al. [82]	Circ_0025908	RA-FLS	Cell vitality and proliferation	Circ_0025908/miR-137/HIPK2	Circ_0025908 regulated the miR-137/HIPK2 axis to influence cell vitality and proliferation
Yang et al. [27]	CircRNA_09505	Macrophages, CIA mice	Inflammation and joint damage	CircRNA_09505/miR-6089/AKT1/NF-κB	CircRNA_09505 modulated the miR-6089/AKT1/NF-κB axis to exacerbate inflammation and joint damage in CIA mice
Zhang et al. [83]	Circ_0004712	RA-FLS	Migration	Circ_0004712/miR-633/TRAF6	Circ_0004712 promoted migration *via* miR-633/TRAF6 axis
Yang et al. [84]	CircPTTG1IP	RA-FLS	Proliferation, migration and invasion, as well as apoptosis	CircPTTG1IP/miR-431-5p/FSTL1	CircPTTG1IP promoted RA progression by regulatingmiR-431-5p/FSTL1 axis
Feng et al. [85]	Circ_0088194	RA-FLS	Proliferation, migration, apoptosis, as well as inflammation	Circ_0088194/miR-30a-3p/ADAM10	Circ_0088194 promoted inflammatory response and biological functions through the miR-30a-3p/ADAM10 axis

## lncRNAs and circRNAs as latent diagnostic and prognostic biomarkers

The advancement of high-throughput sequencing and bioinformatics has contributed to the discovery of lncRNAs and circRNAs in RA. Quantities of lncRNAs and circRNAs that are dysregulated in RA cells have been identified, which are utilized as key clinical diagnostic biomarkers in RA.

For example, 5045 differentially expressed lncRNAs were identified *via* a genome-wide microarray analysis of 10 RA patients and 10 healthy controls, among which 2410 lncRNAs were upregulated and 2635 lncRNAs were downregulated [86]. Through transcriptome sequencing (RNA-seq), Long et al. found 341 differentially expressed lncRNAs in peripheral blood mononuclear cells (PBMCs) in three RA patients and normal people [87]. Zhang et al. identified 135 lncRNAs differentially expressed in FLSs of RA patients and normal people [88]. Of those, ENST00000483588 was upregulated; uc004afb.1, ENST00000438399 and ENST00000452247 were downregulated in RA-FLSs. Receiver operating characteristic (ROC) curve analysis was performed to show that these lncRNAs can act as a suitable biomarker for RA diagnosis. Moreover, microarray analysis was used to identify disease activity-associated 683 upregulated and 1,416 downregulated lncRNAs in PBMCs from RA patients, which showed that ENST00000456270 could be a biomarker of RA risk and severity [89]. In another study, Wen et al. analysed the difference between apoptosis- and autophagy-related lncRNAs in PBMCs of three RA patients by high-throughput lncRNA sequencing [90]. After biological validation in 20 RA patients and 20 healthy controls, seven lncRNAs (C5orf17, LINC01189, LINC01006, MAPKAPK5-AS1, DSCR9, MIR22HG and ENST00000619282) were validated as the most significantly differentially expressed lncRNAs, which were correlated with clinical indicators and self-cognitive ability of patients [90].

In 2017, a total of 584 circRNAs (255 upregulated and 329 downregulated circRNAs) differentially expressed were observed in RA patients through the circRNA microarray analysis [91]. Moreover, the differentially expressed circRNAs in RA were screened and validated by the high-throughput analysis and bioinformatics analyses, underscoring the capacity of hsa_circ_0003353 in immunity and inflammation of RA [92]. Through gene microarray technology, Lu et al. obtained 149 upregulated and 250 downregulated circRNAs in PBMCs from RA patients, which showed that lncRNA hsa_circ_101328 has a significant positive correlation with C-reactive protein (CRP) and may be a new marker of RA diagnosis [93]. Alternatively, a recent study by Wen et al. found 165 differentially expressed circRNAs *via* RNA-seq of three RA patients and three healthy controls, further clinical study revealed that hsa circ 0001200, hsa circ 0001566, hsa circ 0003972, as well as hsa circ 0008360 expression levels were in accord with the RNA-seq, which could act as potent biomarkers for RA diagnosis [94]. In comparison, 71 dysregulated circRNAs were identified in RA, and both hsa_circ_0000396 and hsa_circ_0130438 may exhibit a better diagnostic value in RA [95].

In general, there are a large number of aberrantly expressed lncRNAs and circRNAs in RA patients in contrast to normal people. In most studies, the number of lncRNAs and circRNAs was closely related to RA disease activity. Due to the variable abundance and interactions and crosstalk of individual ncRNAs, combinatorially variable series of ncRNAs may be more promising as biomarkers than individual ncRNAs.

## Regulatory roles of ceRNAs in RA

As the vital regulatory mode of gene expression, the lncRNA/circRNA-mediated ceRNA networks are expected to exhibit pleiotropic effects in autoimmune diseases such as RA. ceRNAs have both beneficial (such as suppressing the malignant subtype of RA-FLS) and detrimental (such as promoting inflammatory responses) roles in RA pathogenesis [51,74]. In fact, the lncRNA/circRNA-mediated ceRNA network regulates multiple cellular processes in RA, including proliferation, invasion, inflammation and apoptosis.

### Proliferation, migration and invasion capabilities

Like tumour cells, RA-FLSs also share the properties of biological functions and enhanced resistance to apoptosis. Wang et al. found that lncRNA LINC-PINT is upregulated in TNF-α-induced RA-FLSs, which elevates SOCS1 expression through sponging miR-155-5p, leading to the promotion of the proliferation, migration and invasion [38]. Another study revealed that Linc0238 exacerbates the tumour-like phenotype of FLSs in RA through the miR-590-5p/MAP2K3 axis [42]. In addition, lncRNA HAND2-AS1 binds with miR-143-3p to elevate the levels of TNFAIP3/NF-κB, thus strengthening proliferation, migration and invasion of RA-FLS [45].

As reported, LOC100912373 sponges miR-17-5p to enhance PDK1 expression, thereby facilitating proliferation, migration and invasion of RA-FLS [45]. Also, lncRNA NEAT1 has been revealed to bind with miR-410-3p, thus mediating RA-FLS proliferation, migration and invasion [52]. Furthermore, lncRNA NR-133666 promotes RA progression by acting as a miR-133c sponge and thereby reducing the inhibition of MAPK1 by miR-133c [53]. Wnt signalling is a crucial mediator of cellular activities [55]. lnc RNAS56464.1 promotes RA-FLS malignant subtype by sponging miR-152-3p, thereby activating the Wnt signalling pathway. lncRNAS56464.1 interference inhibits FLS proliferation and reduces the expression of Wnt1, β-catenin, c-Myc, cyclin D1, p-GSK-3β/GSK-3β [55]. Other publications have elucidated the elevation of lncRNA GAS5 [67], lncRNA NEAT1_1 [67] and lncRNA FOXD2-AS1 [68] in RA-FLS. To conclude, the downregulation of these lncRNAs may alleviate the RA-FLS dysfunction, thereby exerting biological activities by serving as ceRNAs.

circRNAs are also implicated in the RA-FLSs biological activities. Hsa_circ_0088036 mediates the RA-FLS biological activities through regulating SIRT1 [70]. Circ_0088194 contributes to RA progression by enhancing MMP2 expression and reducing miR-766-3p [73]. Circ-PTTG1IP suppresses RA-FLS malignant subtypes *via* the miR-671-5p/TLR4 axis [74]. Circ_0008360 plays a protective effect in RA by acting as a miR-135b-5p sponge and downregulating HDAC4 [75].

Inhibiting RA-FLS proliferation, migration and invasion are potential therapeutic strategies for RA. Li et al. proposed that the downregulation of circ_0130438 ameliorates RA by restricting RA-FLS functional properties *via* the miR-130a-3p/KLF9 axis [77]. Also, the downregulation of circASH2L represses RA-FLS tumour-like properties *via* the miR-129-5p/HIPK2 axis [80]. Luo et al. supported that the reduced circMAPK9 retards RA progression *via* the miR-140-3p/PPM1A axis [81]. Similarly, many other ceRNAs, such as circ_0025908/miR-137/HIPK2, circ_0004712/miR-633/TRAF6, circPTTG1IP/miR-431-5p/FSTL1 as well as circ_0088194/miR-30a-3p/ADAM10, have been validated to attenuate RA progression [82,84,85,96].

### Proliferation and apoptosis

Certain ceRNA networks are linked to the viability and apoptosis of RA. Wang et al. in their work suggested that the LINC00152/miR-1270/FOXM1 axis influences the pathogenesis of RA [47]. Depletion of lncRNA ZFAS leads to an inhibition in viability and an enhancement in apoptosis of RA-FLSs *via* the miR-2682-5p/ADAMTS9 axis [48]. Zheng et al. also stated that lncRNA RNA ZFAS1 results in the suppression of the RA process through the miR-296-5/MMP-15 axis [97]. Downregulation of lncRNA GAS5 results in suppressed proliferation and induced apoptosis *via* the modulation of the miR-361-5p/PDK4 axis [62]. CircFADS2 and mTOR were upregulated but miR-498 was downregulated in TNF-α-induced RA-FLS. Moreover, CircFADS2 could mediate mTOR expression *via* binding to miR-498, and CircFADS2 stimulated mitophagy *via* the miR-498/mTOR axis [71]. Furthermore, lncRNA OIP5-AS1/miR-448/TLR3/NF-κB, lncRNA BZRAP1-AS1/miR-1286/COL5A2 and LncRNA XIST/miR-126-3p/NF-κB have been found to strengthen proliferation and restrict apoptosis of RA-FLS [49,54,58].

### Proliferation and inflammation

Some lncRNAs and circRNAs exert proinflammatory and proliferative functions in RA-FLS. LncRNA GAS5 and lncRNA MEG3 were upregulated in RA-FLS. LncRNA GAS5 can sponge miR-222-3p, thus elevating Sirt1 levels [98]. LncRNA MEG3 can act as a ceRNA of miR‐141, thereby activating the AKT/mTOR pathway [41]. Similarly, lncRNA GAPLINC exhibits pro-inflammatory and proliferative effects *via* the miR-382-5p/miR-575 axis [51]. Another study showed that lncRNA OSER1-AS1 is markedly upregulated RA-FLS. Knockdown of lncRNA OSER1-AS1 prevents anti-inflammatory and anti-proliferative capacities by sponging miR-1298-5p [57]. LINC00665 can impede RA-FLS viability and inflammation *via* the miR-122-3p-3p/EIF2AK1 signalling axis [59]. Additionally, lncRNA-H19 acts as a miR-124a sponge and subsequently downregulates CDK2 and MCP-1 levels to facilitate RA-FLS viability and inflammation, leading to RA progression [60]. LncRNA ZNF667-AS1 facilitates RA-FLS viability and inflammation through the miR-523-3p/JAK/STAT axis [61]. LncRNA HOTTIP influences RA-FLS proliferation and inflammation through the miR-1908-5p/STAT3 axis [63]. Moreover, lncRNA NEAT1 in exosomes of RA stimulates proliferation and inflammation *via* the miR-23a/MDM2/SIRT6 axis [50].

Zhi et al. recently revealed that the reduction of circ_AFF2 mediates the miR-375/TAB2 axis to impede RA-FLS proliferation and inflammation [72]. Geng et al. proposed that circ_0088036 contributes to an amelioration of RA progression through blocking miR-326 activity and elevating FZD4 expression [76]. Wang et al. pointed out that circ_0088036 results in an exacerbation of RA *via* the downregulation of miR-1263 and upregulation of REL [78]. In addition, the NF-κB-regulated genes are also vital in the process of invasiveness and inflammation. For instance, Yang et al. first stressed that downregulation of circRNA_09505 attenuates RA progression *via* the miR-6089/AKT1 axis and the modulation of the NF-κB pathway [27]. Additionally, many other ceRNAs, such as lncRNA SNHG14/miR-17-5p/MINK1/JNK [65], lncRNA RNA XIST/let-7c-5p/STAT3 [66] and circ-AFF2/miR-650/CNP [79], have been validated to participate in the RA-FLS viability and inflammation.

### Apoptosis and inflammation

Inflammation and apoptosis are critically important in RA pathogenesis. As described, lncRNA PVT1 was observed to mediate inflammatory responses in RA-FLS by serving as a miR-543 sponge and positively modulating SCUBE2 expression [43]. TLR4, as the main receptor impacting the NF-κB activation, could influence RA progression. Yan et al. addressed that *via* the modulation of the TLR4/miR-6089 axis, lncRNA HIX003209 evoked an inflammatory response in RA [44]. Except that, the signalling axis of LINC01197/miR-150/THBS2 [39] and lncRNA CASC2/miR-18a-5p/BTG3 [56] have been elucidated to intensify inflammation and diminish apoptosis of RA-FLS.

## Methods for characterization of ceRNA interactions

Several bioinformatic tools and genomics databases can be utilized for the construction of a lncRNA/circRNA-miRNA-mRNA network. Different databases have their own prediction rules and characteristics, which leads to different prediction results, and therefore a combination of various databases is needed to give robust information.

Five main algorithms (TargetScan, RNA22, miRanda, PicTar and PITA) are adopted for the prediction of potential miRNA targets, which is helpful for the miRNA-sponge interactions and ceRNA relationships [99–103]. Furthermore, the open-source database StarBase v2.0 (http://starbase.sysu.edu.cn/) provides the CLIP-Seq data to experimentally support the interaction networks of miRNA-mRNA and miRNA-lncRNA [104]. This database incorporates the gene expression data (AGO-CLIP and small RNA-seq data), which increases the reliability of the lncRNA/circRNA-miRNA relationship predictions [105]. Similarly, many databases can be implemented to probe into the interacting miRNAs of lncRNAs. LncCeRBase is a relatively small database that consists of 432 lncRNA-miRNA-mRNA interactions [106]. LncACTdb 2.0 is a comprehensive database that offers comprehensive information on ceRNAs in varying species and diseases [107].

In addition to conducting database predication, the functions of the lncRNA/circRNAs sponging with its target miRNA need to be verified by experiments. First, we need to identify the target lncRNA/circRNA and its functional phenotypes and the clinical diagnostic and prognostic value in diseases. Second, RNA-FISH and nuclear-cytoplasmic separation experiments were chosen to verify whether lncRNA/circRNA mainly located in the cytoplasm, was facilitated to determine whether it can regulate miRNAs at the posttranscriptional level [108]. Also exciting is that in cells and tissues, the FISH assay also can evaluate the colocalization of lncRNA/circRNA with miRNAs. But, it is important to note that circRNA is conserved and stable, and formed through back-splicing events of precursor mRNA, which should avoid recognition of their cognate linear RNAs [109]. Third, the dual-luciferase reporter gene assay has been widely applied to validate human miRNA targets [110]. The luciferase reporter vector wild-type (WT)-lncRNA/circRNA is established by the WT and mutant type (MUT) sequences of lncRNA/circRNA containing the binding sites of miRNA inserting into the pGL3 vector (Promega Corporation) [110]. Transfection with miRNA mimics diminished the WT luciferase reporter activity; yet, the transfection failed to diminish the MUT luciferase reporter activity [110]. Forth, the co-immunoprecipitation of lncRNA/circRNA and miRNA with anti-AGO2 could suggest that lncRNA/circRNA is observed in RNA-induced silencing complexes containing AGO2, possibly through the interaction with miRNA, which further validates lncRNA/circRNA’s miRNA sequestering activity [111]. More importantly, direct interaction between lncRNA/circRNA and miRNA was confirmed by luciferase activity and RIP assays, this finding strongly supported the ceRNA theory that lncRNA/circRNA could compete for miRNA [112]. Finally, an RNA pull-down assay with biotinylated probes can be designed specifically for the lncRNA/circRNA-miRNA [113]. After addressing the RNAs obtained after the enrichment, the interacting miRNA-lncRNA/circRNA molecules are elucidated by mass spectrometry analyses [114].

## ceRNA involved in TCM treatment of RA

TCM has attracted more and more attention owing to its advantages of safety and fewer adverse reactions [75]. The use of herbs in treating RA has a history of thousands of years in many Asian countries, and curative effects have been proven by both clinical applications and experimental research [18,115]. As is well known, herbal medicine can function through multiple targets and multiple pathways [116,117]. Herb medicine is anti-rheumatic and possesses diverse pharmacological actions, such as regulation of anti-inflammatory, analgesic and immunomodulatory, inhibits hyperplasia of synovial cells, and suppresses angiogenesis [118,119]. It is still noteworthy that herbal medicines and their monomer show promising effects on the inhibition of synovial hyperplasia, and the specific mechanisms are primarily realized *via* the regulation of ceRNA. We summarized that TCM exerts its therapeutic effect in RA by regulation of ceRNAs, as detailed in Table 2.

**Table 2.** Traditional Chinese medicine (TCM) exerts its therapeutic effect on rheumatoid arthritis (RA) by regulating the competitive endogenous RNAs (ceRNAs).

Reference	LncRNA/CircRNA	Model	Pathophysiological mechanism	Axis	Effects	TCM
Wang et al. [120]	lncRNA uc.477	RA-FLS, CIA mice	Inflammation	lncRNA uc.477/miR-19b	Chinese herbal formula HQT functions in RA therapy *via* its regulation of lncRNA uc.477 and miR-19b	Huayu Qiangshen Tongbi formula
Yang et al. [121]	lncRNA-NR024118	Balb/C female mice, MH7A	Inflammation	lncRNA-NR024118	Shikonin inhibits inflammatory reaction in RA-FLS *via* mediating lncRNA-NR024118	Shikonin
Ma et al. [122]	Circ-FAM120A	RA-FLS	Proliferation, mobility and inflammation and triggered cell cycle	Circ-FAM120A/ miR-671-5p/MDM4	Paeoniflorin suppresses the RA process *via* modulating the Circ-FAM120A/miR-671-5p/MDM4 axis	Paeoniflorin
Zhang et al. [123]	lncRNA ENST00000494760	CIA mouse model, MH7A	Inflammation and bone destruction	lncRNA ENST00000494760/ miR-654-5p/C1QC	A novel ceRNA regulatory axis is inference with the individual differences in response to tripterysium glycosides in RA patients	tripterysium glycosides
Zhou et al. [124]	lncRNA WAKMAR2	RA-FLS	Proliferation, invasion and inflammation	lncRNA WAKMAR2/miR-4478/E2F1/p53	Therapeutic effects of (5 R)-5-Hydroxytriptolide on RA-FLS *via* lncRNA WAKMAR2/miR-4478/E2F1/p53 axis	(5R)-5-hydroxytriptolide
Zhang et al. [125]	CircHIPK3	RA-FLS, HDMEC	Angiogenesis and inflammation	CircHIPK3/miR-149-5p/FOXO1/VEGF	Arsenic trioxide impedes angiogenesis in RA by the circHIPK3/miR-149-5p/FOXO1/VEGF axis	Angiogenesis
Pan et al. [126]	lncRNA MALAT1	RA-FLS	Apoptosis	lncRNA MALAT1	Quercetin promotes RA-FLS apoptosis by elevating lncRNA MALAT1	Quercetin
Jiang et al. [127]	LOC100912373	SD mouse, RA-FLS	Proliferation	LOC100912373/miR-17-5p/PDK1	Astragaloside mediates the lncRNA LOC100912373/miR-17-5p/PDK1 axis to suppress the proliferation of RA-FLS	Astragaloside
Li et al. [128]	lncRNA GAS5	RA-FLS, RA syno*via*l tissues	Apoptosis	lncRNA GAS5	Tanshinone IIA evokes RA-FLS apoptosis *via* enhancing lncRNA GAS5	Tanshinone IIA
Piao et al. [129]	lncRNA RP11-83J16.1	RA-FLS, CIA mice	Proliferation, invasion and inflammation	lncRNA RP11-83J16.1/URI1/β-catenin	Triptolide exhibits a therapeutic effect in CIA rats *via* the lncRNA RP11-83J16.1/URI1/β-catenin axis	Triptolide
Wen et al. [130]	hsa-circ-0003353	RA-FLS	Cell growth and inflammatory response	hsa-circ-0003353/miRNA-31-5p/CDK1	Triptolide modulates the hsa-circ-0003353/microRNA-31-5p/CDK1 axis to retard RA-FLS growth and inflammatory response	Triptolide
Wen et al. [131]	lncRNA ENST00000619282	RA-FLS	Apoptosis and inflammation	lncRNA ENST00000619282	Triptolide decreases ENST00000619282 to stimulate the apoptosis and reliefs the inflammation of RA-FLS	Triptolide
Wang et al. [132]	circ_0015756	RA-FLS, CIA-FLS	Inflammation	circ_0015756/CUL4B/Wnt	The RA pathogenesis is delayed by the Traditional Chinese medicine compound Huangqin Qingre Chubi Capsule *via* the CUL4B/Wnt Pathway	Huangqin Qingre Chubi Capsule
Wang et al. [133]	circRNA 0003353	RA-FLS	Inflammatory response and migration	circRNA 0003353	Triptolide inhibits inflammatory reaction and migration capacity of RA-FLS by the circRNA 0003353/JAK2/STAT3 axis	Triptolide
Pan et al. [134]	LncRNA OIP5-AS1	SD mouse, RA-FLS	Proliferation	LncRNA OIP5-AS1/miR-410-3p/Wnt7b	Total Saponins of Radix Clematis impacts RA-FLS proliferation *via* the modulation of the lncRNA OIP5-AS1/miR-410-3p/Wnt7b axis	Total Saponins
Wen et al. [135]	lncRNA MAPKAPK5-AS1	RA-FLS	Apoptosis and inflammation	lncRNA MAPKAPK5-AS1	Xinfeng Capsules promote RA-FLS apoptosis and attenuate inflammation by regulating lncRNA MAPKAPK5-AS1	Xinfeng capsules
Duan et al. [136]	lncRNA NEAT1	RA-FLS	Proliferation and apoptosis	lncRNA NEAT1/miR-17-5p/STAT3	Tetrandrine-induced reduction of lncRNA NEAT1/STAT3/miR-17-5p axis inhibits RA progression	Tetrandrine

### lncRNA/circRNA involved in Chinese medicine monomer treatment of RA

*Tripterygium wilfordii* Hook.f., also called Leigongteng in TCM, is a commonly used anti-rheumatic herbal drug [137]. Triptolide (TPL) is a diterpene lactone epoxide compound extracted from *Tripterygium*, and it possesses diverse biological profiles, such as anti-fertility, anti-tumour, anti-inflammatory, as well as immunosuppressive activities [138,139]. TPL inhibits RA-FLS proliferation, invasion and inflammation by suppressing the levels of TNF-α, IL-1β, IL-6, MMP-3 and MMP-9, exhibiting a therapeutic role in collagen-induced arthritis (CIA) rats [129]. However, these effects were reversed by lncRNA RP11-83J16.1 overexpression [129]. ENST00000619282 expression was elevated both in RA-PBMCs and RA-FLS, while ENST00000619282 was significantly decreased following treatment with TPL [131]. In addition, ENST00000619282 showed a close clinical correlation with the disease activity. Furthermore, TPL exerts an anti-inflammatory and pro-apoptotic function that was reversed by overexpression ENST00000619282. The same conclusion could be drawn from another study. TPL exerts anti-inflammatory and anti-migratory effects in RA-FLS through the circRNA 0003353/JAK2/STAT3 signalling pathway [133].

Other Chinese medicine monomers were also reported to exhibit a potential role in treating RA *via* lncRNAs. For instance, shikonins have been reported to confer an anti-inflammatory role against RA. In a study conducted by Yang et al. shikonin inhibits RA-FLS inflammatory reaction *via* lncRNA-NR024118 [121]. Additionally, it has been found that tanshinone IIA promotes RA-FLS apoptosis by elevating lncRNA GAS5 [128]. Moreover, Fang et al. also found that quercetin contributes to induction of RA-FLS apoptosis by upregulating lncRNA MALAT1 [128].

### lncRNA/circRNA involved in TCM compound treatment of RA

Xinfeng Capsule (XFC) is a TCM prescription that is commonly applied in RA therapy, and which is composed of four TCM components: *Radix astragali*, *Coicis semen*, *T. wilfordii* and *Centipedes* [140]. A recent review demonstrates the effectiveness and safety of XFC for RA therapy *via* meta-analysis. A multicenter, parallel, placebo-controlled, double-blind and randomized controlled trial (RCT) involving 304 patients with RA from China showed that XFC can effectively reduce joint pain and improve laboratory indicators, which was found to be comparable to leflunomide [141]. The level of lncRNA MAPKAPK5-AS1 in the RA-PBMCs and RA-FLS were down-regulated and can mediate RA-FLS inflammation and apoptosis [135]. Interestingly, restored lncRNA MAPKAPK5-AS1 can reverse the effect of Xinfeng Capsules (XFC) on RA inflammation and apoptosis, which indicated that lncRNA MK5-AS1 participated in RA treatment with XFC [135].

Huayu Qiangshen Tongbi formula (HQT) is utilized for RA treatment by dissipating blood stasis, activating blood circulation, as well as dispelling pathogenic cold, wind and wet. There has been a study indicating that lncRNA uc.477 has a direct regulatory effect on the expression of miR-19b in RA [120]. Importantly, HQT treatment normalized the lncRNA uc.477 and miR-19b levels in RA-FLS and the CIA mice model. Thus, lncRNA uc.477 could be a latent therapeutic marker for HQT on RA and its therapeutic mechanism may be through the upregulation of miR-19b.

### ceRNA involved in Chinese medicine monomer treatment of RA

Many ceRNA networks were linked to the treatment of *T. wilfordii* in RA. Zhang et al. suggested that the ceRNA network (lncRNA ENST0000494760/miR-654-5p/C1QC) is confirmed by the results obtained from microarray and was deemed to be a biomarker for RA response to Tripterysium Glycosides Tablets (TGT) by clinical cohort, *in vitro* and *in vivo* experiments [123]. Circ0003353 was confirmed as a key circRNA that reacted to inflammation and immunity in RA. It was found that circ0003353 sponged miR-31-5p to upregulate CDK1 and thus promote RA-FLS proliferation and inflammation. Interestingly, circ0003353/miR-31-5p/CDK1 axis could reverse the effect of TPL on RA -FLS through a series of rescue and gain-of-function experiments [130]. (5 R)-5-hydroxytriptolide (LLDT-8), as a novel analogue of TPL, is both qualified and optimized in structure, and possesses better immunosuppressive activities and lower toxicities than TPL. The LLDT-8-induced elevation of lncRNA WAKMAR2 induced was the most remarkable in RA-FLS and restored WAKMAR2 evoked cell viability, invasion, as well as inflammation in RA-FLS. Mechanistically, it has been proven that WAKMAR2 plays a ceRNA role in regulating E2F1 expression by competitively binding to miR-4478, which can be regulated by LLDT-8 [142]. Thus, it is important to consider the ceRNA axis as a potential therapeutic target for *T. wilfordi*i in RA.

Of course, other studies have unveiled the effect of Chinese medicine monomer treatment on RA through the ceRNA axis. For example, In a study conducted by Ma et al. paeoniforin mediated the circ-FAM120A/miR-671-5p/MDM4 pathway to impede RA-FLS viability and inflammation and trigger cell cycle arrest [122]. Additionally, one study found that astragaloside regulates the LOC100912373/miR-17-5p/PDK1 axis for the suppressed FLS proliferation in rats with RA [143]. Pan et al. also found that total saponins of radix clematis modulated RA-FLS *via* the OIP5-AS1/miR-410-3p/wnt7b axis [134]. Furthermore, arsenic trioxide harbours a protective function on RA-FLS and CIA synovium through blocking the circHIPK3/miR-149-5p/FOXO1/VEGF module [125]. Another publication by Duan et al. also elucidated that tetrandrine downregulates NEAT1 expression, mechanistically, NEAT1 exerts ‘sponge-like’ effects on specific miR-17-5p to affect miR-17-5p binding to target gene STAT3, causing restricted RA-FLS viability and proliferation [144]. Thus, the above studies lay a basis for an effective treatment approach for RA.

### ceRNA involved in TCM compound treatment of RA

Huangqin Qingre Chubi Capsule (HQC) is a prescription for RA therapy, which is currently used as an in-hospital preparation at the First Affiliated Hospital of Anhui University of Chinese medicine [145]. The study on peripheral blood mononuclear cells of 24 patients with RA from China showed that HQC drug-containing serum could activate Adenosine 5′-monophosphate (AMP)-activated protein kinase (AMPK) and Forkhead box protein O3a (FoxO3a) proteins in PBMCs of patients with RA and improve the state of oxidative stress in patients with RA [145]. Clinical studies have shown that HQC can significantly reduce indicators of disease activity and that it has a good therapeutic effect on decreasing joint pain and improving joint function [146]. Circ_0015756 expression was upregulated both in RA-FLS, synovium of CIA mice, as well as CIA-FLS. As reported, circ_0015756 expedited the inflammation and viability of RA by modulating the miR-942-5p/CUL4B/Wnt axis. HQC can attenuate joint damage in CIA mice and inhibit inflammation and proliferation of RA-FLS, which is associated with its interference with the effects of the circ_0015756/miR-942-5p/CUL4B axis [132]. The discovery of this axis offers a novel ceRNA mechanism for RA and provides a basis for HQC’s functions in RA through multimolecular, multitarget and multi-pathway.

## Conclusion and prospects

In the last decade, substantial progress has been made in identifying the genetic basis of RA thanks to the generation of several molecular tools and experimental studies. However, the available clinical therapeutic strategies for RA are still unsatisfactory. ncRNAs are now considered a hot topic of scientific research due to their great potential. Available evidence suggests that ceRNAs can regulate inflammation and autoimmunity. It has been elucidated that lncRNA/circRNA, miRNA and mRNA can play an integral regulatory role in the pathological process of RA in a ceRNA pattern. Specifically, ceRNAs are involved in proliferation, invasion, inflammation and apoptosis phenotypes in RA. Altering lncRNA/circRNA levels to affect target gene levels to reverse RA is promising. In addition, low toxicity and multi-targeted herbal medicines affect ceRNAs at the epigenetic level, which may provide a new reference for the treatment of RA.

Although the role of ceRNAs in RA is becoming increasingly evident, their specific mechanisms in RA progression need to be further explored. Existing studies on the regulation of ceRNA by herbal medicine are still limited to preclinical investigations at the molecular, cellular and animal model levels, which are challenging and promising to translate into clinical practice. Meanwhile, how to ensure therapeutic efficacy and safety, and prevent off-target effects, need to be considered.

In the future, studies on the interactions of ncRNA, ceRNA and RA should lay much attention on the following aspects. Initially, the construction of complex regulatory ceRNA network models with single lncRNA or circRNA modifications in RA is required. Focus on the improvement of the new network model to obtain and develop novel targets or treatment strategies for RA is certainly warranted. Second, in future studies, the sample size must be expanded to improve the reliability of the findings. Third, further validation with *in vivo* studies is critical for the development of ceRNA-targeted therapy in RA. Forth, in combination with the cells-secreted exosomes or vesicles, the feasibility and safety of RNA-modifying factors wrapped in exosomes is the basis of RA progression. Fifth, it is currently unknown how to effectively control the lncRNA/circRNA levels in target cells. m6A modification was abundant in many circRNAs and lncRNAs, and this kind of methylation modification could drive circRNA and lncRNA translation. Therefore, it is also important to explore the relationship between epigenetic modification and ceRNAs. Finally, TCM is known as multi-compound and multitarget medicines for wide application in RA through multiple targets, pathways, as well as links. UPLC-Q/TOF-MS analysis is a fact and useful technology for identifying TCM complex chemical compounds, which contribute to identifying the main effective ingredient of TCM to exhibit a potential role in treating RA through ceRNA.

In summary, this paper reviews the regulation of RA progression by lncRNA/circRNA-mediated ceRNA patterns involving the regulation of multiple phenotypes of proliferation, migration, apoptosis and inflammation, which provides new avenues for the exploration of autoimmune diseases including RA. Likewise, this review highlights that herbal medicines and their components can treat RA *via* ceRNAs, pointing to some directions for the clinical application of the aforementioned herbal medicines as anti-RA agents.
